# Supplementary material for: Exploring the use of the Psychological Characteristics of Developing Excellence (PCDEs) in younger age groups: First steps in the validation process of the PCDE Questionnaire for Children (PCDEQ-C)
Source: PLoS One. 2021 Nov 18;16(11):e0259396. doi: 10.1371/journal.pone.0259396 (PMC8601555; doi:10.1371/journal.pone.0259396)
Supplement: S1 Table — (PDF) [file pone.0259396.s001.pdf]

Supplementary Material 1

**S1 Table**. Distribution per age and sex, type of sport and level.

| | | Team sports | | | Individual sports | | | Total |
|---|---|---|---|---|---|---|---|---|
| **Age** | **Sex** | **Recreational** | **Competitive** | **Total** | **Recreational** | **Competitive** | **Total** | |
| **7** | **Male** | 1 | 14 | 15 | 10 | 4 | 14 | 29 |
| | **Female** | 1 | 3 | 4 | 18 | 0 | 18 | 22 |
| **8** | **Male** | 4 | 13 | 17 | 5 | 11 | 16 | 33 |
| | **Female** | 0 | 1 | 1 | 23 | 34 | 57 | 58 |
| **9** | **Male** | 7 | 24 | 31 | 12 | 10 | 22 | 53 |
| | **Female** | 5 | 7 | 12 | 25 | 46 | 71 | 83 |
| **10** | **Male** | 7 | 38 | 45 | 8 | 19 | 27 | 72 |
| | **Female** | 5 | 6 | 11 | 35 | 30 | 65 | 76 |
| **11** | **Male** | 10 | 39 | 49 | 7 | 32 | 39 | 88 |
| | **Female** | 3 | 9 | 12 | 19 | 28 | 47 | 59 |
| **12** | **Male** | 8 | 48 | 56 | 6 | 36 | 42 | 98 |
| | **Female** | 8 | 17 | 34 | 34 | 43 | 77 | 102 |
