# Supplementary material for: Exploring the use of the Psychological Characteristics of Developing Excellence (PCDEs) in younger age groups: First steps in the validation process of the PCDE Questionnaire for Children (PCDEQ-C)
Source: PLoS One. 2021 Nov 18;16(11):e0259396. doi: 10.1371/journal.pone.0259396 (PMC8601555; doi:10.1371/journal.pone.0259396)
Supplement: S2 Table — (PDF) [file pone.0259396.s002.pdf]

Supplementary Material 2

S2 Table. Items per factor of the PCDEQ-C

Factor	Items
Factor 1<br>Performance Worries<br>(N= 16)	When things don't work out for me I worry about my future.<br>I often lie awake at night and keep thinking about the same things.<br>Daily problems can often make me feel sad.<br>My sleep is often disturbed by troubling thoughts in my head.<br>Even small setbacks can make me lose my focus.<br>Sometimes, when I don't succeed in something. I hate that I cannot control the result.<br>I am often worried that bad things will happen.<br>I often keep on thinking about mistakes I have made, which doesn't do my performance any good.<br>If I am nervous, I find it difficult to overcome this feeling when I am performing.<br>If I don't spend all my time and attention on my sport, my performance will suffer.<br>If something goes wrong I find it hard to see how to continue afterwards.<br>I often find it difficult to talk to other people about things that are bothering me.<br>If something unexpected happens, I find it very hard to adapt myself to that.<br>I find it hard to convince myself to overcome problems.<br>If I make a mistake I dwell on it and can't see the big picture.<br>I often feel nervous.
Factor 2<br>Social support<br>(N = 9)	I think that asking someone else for help is a sign of weakness.<br>When I have a problem, I don't have anyone I can turn to for help.<br>I often forget appointments or timings.<br>Compared to my teammates I succeed less often in completing a hard training session.

	I find it difficult to get myself motivated.<br>I am no longer interested in hanging out with my training group.<br>I sometimes feel unhappy without really knowing why.<br>When I have a bad practice or competition, the people I consider important are often disappointed in me.<br>I don’t like asking other people for help and advice.
Factor 3<br>Imagery and Active Preparation<br>(N = 13)	When I prepare for learning skills or playing games, I use imagery.<br>I use imagery to improve my performance.<br>I have a well thought-out plan to become an elite athlete.<br>I imagine in advance how I would deal with setbacks.<br>I regularly imagine what it feels like to have a good performance.<br>I regularly think through and plan for exactly what I want to achieve.<br>I like to try things out in my head first.<br>I repeat my routine in my head so that I really know what to pay attention to.<br>I take time to make clear what has been asked of me.<br>I am someone who runs through things over and over again in my head.<br>Before I start a routine I imagine executing it.<br>Every training session I go through my routines in my head as well as physically doing them.<br>Before I arrive at the competition I already go through what I am supposed to do during the competition in my head.
Factor 4<br>Adverse Response to Failure<br>(N = 7)	When I have eaten something I sometimes feel guilty because eating changes my body shape<br>When I don’t succeed in something, I think that people won’t be interested in me anymore.<br>I am worried that I am getting too heavy.<br>When things are not going well, I am worried about what other people will think.

	When I fail, people are less interested in me.<br>When I don't succeed in something I am mostly worried about what others will think about me.<br>If I have a bad practice or competition, I am afraid I'll never be able to make it.
Factor 5<br>Self-Directed Control and Management<br>(N = 6)	I often do something without thinking about other ways of doing it.<br>I usually blame a failure on other people or circumstances.<br>If something unexpected happens, I find it very hard to adapt myself to that.<br>I often do things I know I shouldn't.<br>I do some things that aren't good for me because I like them.<br>I find it difficult to stop bad habits.
