# Supplementary material for: Exploring the use of the Psychological Characteristics of Developing Excellence (PCDEs) in younger age groups: First steps in the validation process of the PCDE Questionnaire for Children (PCDEQ-C)
Source: PLoS One. 2021 Nov 18;16(11):e0259396. doi: 10.1371/journal.pone.0259396 (PMC8601555; doi:10.1371/journal.pone.0259396)
Supplement: S3 Table — (PDF) [file pone.0259396.s003.pdf]

Supplementary Material 3

Comparison of the factor structure of the original PCDEQ-C (Hill et al., 2019) and the current factor structure.

S3 Table. Comparison of the PCDE factors for the PCDEQ-C and PCDEQ-2.

	**PCDEQ-C**	**PCDEQ-2**
**Factor 1**	Adverse Response to Failure (-)	Adverse Response to Failure (-)
**Factor 2**	Imagery and Active Preparation (+)	Imagery and Active Preparation (+)
**Factor 3**	Self-Directed Control and Management (+)	Self-Directed Control and Management (+)
**Factor 4**	Performance Worries (-)	Perfectionistic Tendencies (+/-)
**Factor 5**	Seeking and Using Social Support (+)	Seeking and Using Social Support (+)
**Factor 6**		Active Coping (+)
**Factor 7**		Clinical Indicators (-)
