# Supplementary material for: Exploring the use of the Psychological Characteristics of Developing Excellence (PCDEs) in younger age groups: First steps in the validation process of the PCDE Questionnaire for Children (PCDEQ-C)
Source: PLoS One. 2021 Nov 18;16(11):e0259396. doi: 10.1371/journal.pone.0259396 (PMC8601555; doi:10.1371/journal.pone.0259396)
Supplement: Attachment — Submitted filename: Response to reviewers.docx [file pone.0259396.s004.docx]

**Rebuttal letter**

Dear editor and reviewer,

We are pleased to get the opportunity to revise our manuscript. We would like to thank you and the reviewer for the insightful comments and constructive feedback. In this rebuttal we will respond to each comment and provide a point by point description of how each comment was addressed in the manuscript. The reviewer can follow the changes made in the manuscript, the lines described in the document below are based on the Revised Manuscript with Track Changes.

No references were removed in the revised manuscript, but two references were added:

Munroe-Chandler KJ, Hall CR, Fishburne GJ, Strachan L. Where, when, and why young athletes use imagery: An examination of developmental differences. Research Quarterly for Exercise and Sport. 2007;78(2):103-16.

Uphill MA, Lane AM, Jones MV. Emotion Regulation Questionnaire for use with athletes. Psychology of Sport and Exercise. 2012;13(6):761-70.

**Reviewer 1**

5. Review Comments to the Author
Dear authors,
The work carried out represents an important contribution to the field of knowledge. However, in the opinion of the reviewer, a series of modifications is necessary for the paper to be published.
Thanks for your effort. The research carried out is adequate and well justified. The keywords are well selected.

Thank you for this positive feedback.

INTRODUCTION

Line 38.- The year is missing in Gagné

Thank you for pointing this out, we now also included the year.

Line 47 and 48.- “Researchers have repeatedly recognized psychology as a key determinant in TD 48 (4-6)” It is recommended to include references from MacNamara and Collins 2011 (PCDEQ).

We thank you for this comment, but this reference was already added in the subsequent sentence (reference number 7), to further clarify our point.

Lines 45-47: Researchers have repeatedly recognised psychology as a key determinant in TD (4-6). In fact, research has suggested for a while that psychology is an even more important factor in TD than in eventual adult performance (7).

Reflection: Can talent be detected with 7-year-old athletes? Are these children ready for a self-report assessment? Such a low age range is not adequately justified for self-report psychological tests. Most of the tests used in Sports Psychology are for athletes aged 12 years or older due to: intellectual abilities developed close to adulthood; development of self-concept (among others).

We agree with the reviewer that 7 years is young, but in several sports (especially early-specialisation sports such as gymnastics) athletes are indeed already identified and selected at these young ages. This identification is mostly based upon physical and/or sport-specific tests.

We used a psychological questionnaire to assess psychological characteristics in the talent development pathway. However, to clarify, the PCDEQ-C is not used as a talent selection or identification tool at any age. The questionnaire should only be used as a source of formative feedback.

Lines 422-427: The present study provides a first step in the validation process of a formative assessment tool for the developmental psychological characteristics in athletes between 7 and 13 years old, called the PCDEQ-C. Rather than a talent selection tool, this questionnaire should be used as a formative assessment tool to monitor, develop, and design the psychological characteristics needed during the entire TDE pathway of a young, potential athlete.

As the reviewer points out, these children are indeed young for self-assessment. However, research has been pointing out that even young children are cognitively ready to use self-report methods for mental health (Sharp et al., 2006; Chorpita et al., 2000; Patalay et al., 2014), self-esteem and self-concepts (Butler and Gasson, 2005) or even imagery use (Munroe-Chandler et al., 2007), when the language is age-adapted. Therefore, we believe the cognitive development of young children is mature enough to not only assess their own psychological characteristics, but also to learn how to strengthen the underlying psychological skills. However, it would be more advisable to use cognitive maturity cut-offs, rather than chronological age cut-offs, to examine if children are ready to fill out a psychological questionnaire. We have now added this to the limitation section:

Lines 392-395: Some limitations regarding the validation process of the PCDEQ-C should be addressed. First, the PCDEQ-C is now based on age-cut offs, although it could be interesting to investigate if cognitive maturity level could be a better indicator for understanding and using the PCDEs.

Furthermore, during the validation process of the questionnaire, we checked if all items were age-appropriate and, following feedback, some changes were made. We also recognised that supervision or the presence of an independent adult is advisable when young children fill out the questionnaire

Lines 135-138: First, the appropriateness of the English items in the original questionnaire (the PCDEQ2) was checked by the Belgian-British panel. Items that were too difficult for 8-year-olds were rephrased in English. This only led to rewriting 3 items, specifically because of the term ‘visualisation’.

Lines 365-369: During testing, it was observed that filling-out the questionnaire was difficult for the youngest participants. Although they understood the items in the questionnaire, they were more comfortable providing answers when a researcher read the items out loud. We advise to complete the assessment of the PCDEQ-C in a quiet room, with a one-on-one guidance, to make the responses of the young athletes as accurate as possible.

References:

- Sharp C, Goodyer IM, Croudace T: The Short Mood and Feelings Questionnaire (SMFQ): a unidimensional item response theory and categorical data factor analysis of self-report ratings from a community sample of 7-through 11-year-old children. J Abnorm Child Psychol. 2006, 34: 379-391.
- Chorpita BF, Yim L, Moffitt C, Umemoto LA, Francis SE: Assessment of symptoms of DSM-IV anxiety and depression in children: a revised child anxiety and depression scale. Behav Res Ther. 2000, 38: 835-855. 10.1016/S0005-7967(99)00130-8.
- Patalay, P., Deighton, J., Fonagy, P., Vostanis, P., & Wolpert, M. (2014). Clinical validity of the Me and My School questionnaire: a self-report mental health measure for children and adolescents. Child and Adolescent Psychiatry and Mental Health, 8(1), 1-7.
- Butler, R. J., & Gasson, S. L. (2005). Self esteem/self concept scales for children and adolescents: A review. Child and Adolescent mental health, 10(4), 190-201.
- Munroe-Chandler, K. J., Hall, C. R., Fishburne, G. J., & Strachan, L. (2007). Where, when, and why young athletes use imagery: An examination of developmental differences. Research quarterly for exercise and sport, 78(2), 103-116.

Specify that the PCDEQ questionnaire is a self-perception and self-report questionnaire, not based on an objective analysis of the reality that surrounds the athlete (evaluation by experts, such as coaches; evaluation of the family, parents or guardians; not based on aptitude tests) .

We agree with the reviewer that this is indeed a self-perception and self-report questionnaire. Furthermore, filling out this questionnaire only shows a snapshot of that particular stage on the non-linear and dynamical talent developmental pathway. Therefore, we encourage using the PCDEQ-C as a formative assessment tool only, not as a talent selection or identification tool. The questionnaire should be used in a talent development context, where it is part of the triangulation process (Hill et al., 2019). Using the questionnaire, next to other measures, such as behavioural observations, coaches’ opinions, dialogue with family or guardians, should give a better view of the athlete’s profile.

We have now made changes, to clarify this further in the method and discussion section:

Lines 187-189: The questionnaire consisted of 87 items on a 6-point Likert scale, ranging from 1 (“very unlike me”) to 6 (“very like me”). Notice that this is a self-report and self-perception type of questionnaire.

Lines 395-399: It should also be recognised that the questionnaires regarding the PCDEs are filled-in by the athletes themselves. Next to the general issues with self-report, special care should be given to the competitive nature of talent development environments, where individuals potentially employ impression management strategies when responding to any questionnaire

References:

- Hill, A., MacNamara, Á., & Collins, D. (2019). Development and initial validation of the Psychological Characteristics of Developing Excellence Questionnaire version 2 (PCDEQ2). European journal of sport science, 19(4), 517-528.

Does the test have the capacity to detect inappropriate or maladaptive behaviors such as perfectionism or burnout?

The first PCDE-questionnaire only assessed psychological characteristics that had an adaptive effect on the talent development pathway. However, athletes also need to cope with characteristics that have a maladaptive or dual-effect on the process. That is why these negative psycho-behavioural characteristics were also added in the PCDEQ-2. The items used to assess these maladaptive and dual-effect behaviours, were all directly related to the talent development pathway, and came from qualitative interviews with both coaches and clinicians in the field of talent development. Hill and colleagues (2019) also used items from previously validated psychometric questionnaires (e.g. the Performance Failure Appraisal Inventory; Conroy et al., 2002).

However, the PCDEQ-2 is not built to detect maladaptive behaviour, but rather to alert coaches and athletes that the individual may be more susceptible to use this maladaptive behaviour in their talent development process. When athletes have high scores on maladaptive PCDE factors, such as ‘clinical indicators’, the athlete should be followed-up with a clinical expert and use of other psychometric tools (as is indicated in the Diagnostic and Statistical Manual of Mental Disorders *-*5).

References:

- Hill, A., MacNamara, Á., & Collins, D. (2019). Development and initial validation of the Psychological Characteristics of Developing Excellence Questionnaire version 2 (PCDEQ2). European journal of sport science, 19(4), 517-528.
- Conroy, D. E., Willow, J. P., & Metzler, J. N. (2002). Multidimensional fear of failure measurement: The performance failure appraisal inventory. Journal of Applied Sport Psychology, 14(2), 76-90. doi:10.1080/10413200252907752
- American Psychiatric Association, & American Psychiatric Association. (2013). Diagnostic and statistical manual of mental disorders: DSM-5. Arlington, VA.

Is the adaptation created for both individual and collective sports valid?

The items used in the PCDEQ-2 were originally created for team sports. However, when conducting the cognitive interviews, the items were also checked on usability in individual sports. We concluded that all items were general enough to use in both individual and team sports contexts.

However, earlier studies with the PCDEQ have pointed out that there are differences between type of sports and how the PCDE’s are deployed by the athletes (MacNamara & Collins, 2013). Further research needs to investigate if this is also the case for the PCDEQ-2, especially with the younger athletes.

References:

- Macnamara, A., & Collins, D. (2013). Do mental skills make champions? Examining the discriminant function of the psychological characteristics of developing excellence questionnaire. Journal of sports sciences, 31(7), 736-744.

Isn't it too long a test with 87 items for validation?

We agree that for some athletes, a questionnaire with 87 items can be too long, especially athletes that are less cognitively mature (regardless of whether these athletes are young or old).

In mitigation, the participants could take their own time to fill out the questionnaire and could thus also take a short break after for example one page. We now further clarified this in the method section.

Lines 149-150: Since this is a rather elaborate questionnaire (87 items), a short break after each page was allowed for the participants (more or less after 10 items).

Lines 189-191: Participants had to work individually; however, they were in the same room with their team or class. Here as well, participants completed the questionnaire in their own time and were allowed to take short breaks in between.

The original questionnaire, the PCDEQ-2 with 87 items, was also validated by Hill and colleagues (2019) in two steps. Results from the exploratory factor analysis showed an overall good reliability of α = 0.879. The second step, a discriminant function analysis, also showed a good construct/discriminatory validity, with 72.9% of the participants that were classified correctly. Overall, results from Hill’s study show that, although the questionnaire is lengthy, it is indeed valid in.

References:

- Hill, A., MacNamara, Á., & Collins, D. (2019). Development and initial validation of the Psychological Characteristics of Developing Excellence Questionnaire version 2 (PCDEQ2). European journal of sport science, 19(4), 517-528.

METHOD

164.- “Results Based on the cognitive interviews and back-translation”. To include references.

The reference is already included in lines 145-147, where we explain how the cognitive interviews were conducted:

Lines 143-145: Cognitive interviewing was conducted applying the principles developed by Willis and colleagues (21), using think-aloud protocols, reinterpretations, observations with proactive and reactive verbal probing.

171.The characteristics of the participants are not sufficiently specified. Indicate the type of sport practiced, the weekly practice time, the sporting experience, if they are competitors or not, how many are federated.

The parents were asked to complete a short demographic survey, including questions related to the involvement of organised sports activity of the child during the school year at the time of measurement (i.e., which sport activity and how many practice hours per week).

We have now further elaborated the inclusion criteria used in this study, to clarify that both competitive and recreational athletes were included in this study. Furthermore, different types of sport were used in the study, yet the main sports practiced by the athletes are: football, hockey, badminton, gymnastics and cycling. We have now added these as examples in the method section:

Lines 176-181: However, only individuals who fully completed the questionnaire (a clear answer on each item) and who practiced an organised sport for least 3 hours per week at a recreational or competitive level, were included in this study. This resulted in a total sample of 773 participants (400 girls), with 495 individual athletes (e.g. gymnastics, cycling and badminton) and 278 team sports (e.g. soccer and hockey) athletes.

We also added information about the distribution per age, sex, individual/team sports athlete and competitive/recreational athlete in a table in supplementary material:

Lines 179-182: This resulted in a total sample of 773 participants (400 girls), with 495 individual athletes (e.g. gymnastics, cycling and badminton) and 278 team sports (e.g. soccer and hockey) athletes. A detailed overview of the distribution per age, sex, type of sport and level can be found in S1 Table.

| **S1 Table**. Distribution per age and sex, type of sport and level. | | | | | | | | | | | |
| --- | --- | --- | --- | --- | --- | --- | --- | --- | --- | --- | --- |
|  |  | **Team sports** | | | **Individual sports** | | | **Total** | |  |  |
| **Age** | **Sex** | **Recreational** | **Competitive** | **Total** | **Recreational** | **Competitive** | **Total** | |  | |  |
| **7** | **Male** | 1 | 14 | 15 | 10 | 4 | 14 | | 29 | |  |
|  | **Female** | 1 | 3 | 4 | 18 | 0 | 18 | | 22 | |  |
| **8** | **Male** | 4 | 13 | 17 | 5 | 11 | 16 | | 33 | |  |
|  | **Female** | 0 | 1 | 1 | 23 | 34 | 57 | | 58 | |  |
| **9** | **Male** | 7 | 24 | 31 | 12 | 10 | 22 | | 53 | |  |
|  | **Female** | 5 | 7 | 12 | 25 | 46 | 71 | | 83 | |  |
| **10** | **Male** | 7 | 38 | 45 | 8 | 19 | 27 | | 72 | |  |
|  | **Female** | 5 | 6 | 11 | 35 | 30 | 65 | | 76 | |  |
| **11** | **Male** | 10 | 39 | 49 | 7 | 32 | 39 | | 88 | |  |
|  | **Female** | 3 | 9 | 12 | 19 | 28 | 47 | | 59 | |  |
| **12** | **Male** | 8 | 48 | 56 | 6 | 36 | 42 | | 98 | |  |
|  | **Female** | 8 | 17 | 34 | 34 | 43 | 77 | | 102 | |  |

Since this is only limited sociodemographic information, we do agree with the reviewer that incorporating this type of information is valuable and studies should address and investigate these variables in future in-depth studies. We have added this comment to the limitation section:

Lines 403-410: Since this was only a first step in the validation process, further research is necessary. Further work should investigate the confirmation of the proposed factor structure, in the different contexts as suggested, and with large sample sizes. More types of sport should be included, so future work could also investigate the discriminating ability in type of sport. Other information regarding training history, training intensity, competitive achievements and/or other sociodemographic variables should also be explored.

The distribution of the sample by age and sex is not shown.

We thank the reviewer for pointing this out, and we have now included this as supplementary material, together with information about type of sport and level, as can be seen in the previous question.

Lines 179-182: This resulted in a total sample of 773 participants (400 girls), with 495 individual athletes (e.g. gymnastics, cycling and badminton) and 278 team sports (e.g. soccer and hockey) athletes. A detailed overview of the distribution per age, sex, type of sport and level can be found in S1 Table.

| **S1 Table**. Distribution per age and sex, type of sport and level. | | | | | | | | | | | |
| --- | --- | --- | --- | --- | --- | --- | --- | --- | --- | --- | --- |
|  |  | **Team sports** | | | **Individual sports** | | | **Total** | |  |  |
| **Age** | **Sex** | **Recreational** | **Competitive** | **Total** | **Recreational** | **Competitive** | **Total** | |  | |  |
| **7** | **Male** | 1 | 14 | 15 | 10 | 4 | 14 | | 29 | |  |
|  | **Female** | 1 | 3 | 4 | 18 | 0 | 18 | | 22 | |  |
| **8** | **Male** | 4 | 13 | 17 | 5 | 11 | 16 | | 33 | |  |
|  | **Female** | 0 | 1 | 1 | 23 | 34 | 57 | | 58 | |  |
| **9** | **Male** | 7 | 24 | 31 | 12 | 10 | 22 | | 53 | |  |
|  | **Female** | 5 | 7 | 12 | 25 | 46 | 71 | | 83 | |  |
| **10** | **Male** | 7 | 38 | 45 | 8 | 19 | 27 | | 72 | |  |
|  | **Female** | 5 | 6 | 11 | 35 | 30 | 65 | | 76 | |  |
| **11** | **Male** | 10 | 39 | 49 | 7 | 32 | 39 | | 88 | |  |
|  | **Female** | 3 | 9 | 12 | 19 | 28 | 47 | | 59 | |  |
| **12** | **Male** | 8 | 48 | 56 | 6 | 36 | 42 | | 98 | |  |
|  | **Female** | 8 | 17 | 34 | 34 | 43 | 77 | | 102 | |  |

It is important to specify that the questionnaire refers to your experiences a few weeks ago, and not considering the total time as an athlete.

This is specified in the method section:

Lines 185-186: Participants were asked to fill out a paper version of the questionnaire while thinking about their main sport and their experiences during the past few weeks.

The statistics used are correct.

Thank you!

It is not understood why an exploratory factorial study has not been carried out to observe the resulting number of factors. The criterion of factors with eigenvalues greater than 1 and the analysis of the slope of the factors has not been included.

We decided to use the parallel analysis (PA) in this study to determine the number of factors to be retained. The PA has been demonstrated to be more accurate, leading to less chance of overestimating the number of factors, than the Kaiser criterion (with the eigenvalues) or the scree plot (Velicer & Jackson, 1990; Fabrigar & Wegener, 2012).

Once this was established, EFAs were used to not only give an indication about the number of factors (as the PA does), but also to see which items would relate to which factor. This is further specified in the method section:

Lines 207-222: To examine the factor structure, first the item-to-total correlation was checked, as recommended by Field (25). Items with a correlation below 0.3 were left out of the further analysis (26). Next, a parallel analysis (PA) was performed to suggest the number of factors that would give the best fit. The parallel analysis is considered the “golden standard” to use when determining the numbers of factors (25). Based on the PA, EFA, with an oblique rotation and the minimal residual method, was used with the suggested number of factors but also, offering a second level check, with more or less factors. This approach was followed to end up with the best possible fitting model, since PA often gives an over- or underestimation of the number of factors (27). The model fit measures used for the EFA, were the chi square (χ²) and degrees of freedom (df), χ²/df with the p-value, comparative fit index (CFI), the Tucker-Lewis index (TLI), root mean square error of approximation (RMSEA) and the standardised root mean square residual (SRMR). To have a good model fit, the following criteria were used: χ²/df <3 (27), CFI and TLI >0.90 (28) , RMSEA and SRMR <0.05 (29). All analyses were performed in R (version 3.4.4; Team, 2013); with the Lavaan and Psych R Packages (version 0.5-12 and version 1.9.12 ; respectively) (30, 31).

References:

- Velicer, W. F., & Jackson, D. N. (1990). Component analysis versus common factor analysis: Some further observations. Multivariate Behavioral Research, 25(1), 97-114. doi:10.1207/s15327906mbr2501_12
- Fabrigar, L. R., & Wegener, D. T. (2012). Exploratory Factor Analysis. Oxford: Oxford university Press.

The test with such a different number of factors is not well understood.

We are not entirely sure what the reviewer refers to here. If the remark relates to why we used an exploratory factor analysis with 5, 6, 7, 8 and 9 factors, this is because the parallel analysis suggested 8 factors. Notably, however, even with parallel analyses, under- and overestimation of the number of factors is possible. Therefore, exploratory factor analyses with more and less factors than suggested by the parallel analysis were examined. This is also explained in these lines:

Lines 227-229: The PA suggested that for the 87-items questionnaire, 8 factors should be used. Accounting for the possible under- and overrepresentation of the PA, the EFA was used with 5, 6, 7, 8 and 9 factors.

It is not understood why the reduction of the items was not carried out before the administration of the test, and they are eliminated afterwards.

A reduction before the administration of the test was not necessary, since the questionnaire used was the PCDEQ-2. As we highlight in the introduction, the underlying psychological characteristics used in this questionnaire (the PCDEs) come from thoroughly investigating the literature, gathering expert opinions and a strong base of qualitative investigation. When we started this study, we assumed that the characteristics (or items resembling these characteristics) were the characteristics that are of importance in talent development at the younger ages. Removing items at this point was thus not necessary. However, we did check if all items would be usable or understandable in a younger population.

Lines 135-137: First, the appropriateness of the English items in the original questionnaire (the PCDEQ2) was checked by the Belgian-British panel. Items that were too difficult for 8-year-olds were rephrased in English.

For the exploratory factor analysis, we then first needed to check the correlation between items (Field, 2013). We could only do this once participants had filled out the questionnaire, since the item-to-total correlation is based upon the answers participants give. If items did not correlate well enough (correlation below 0.3), these items were left out for further EFA.

Lines 207-209: To examine the factor structure, first the item-to-total correlation was checked, as recommended by Field (25). Items with a correlation below 0.3 were left out of the further analysis (26).

References:

- Field A. Discovering statistics using IBM SPSS statistics: sage; 2013.

The factors extracted are very far from the theoretical model initially proposed by the PCDEQ questionnaire (it does not include factors related to anxiety, but it does include factors related to coping).

The original theoretical model stems from the first version of the questionnaire, the PCDEQ by MacNamara and Collins (2011). In a second step, items were added by Hill et al (2019) to also include maladaptive and dual-effect characteristics. The factor structure from the PCDEQ-2 is very similar to the original PCDEQ. Coping is for example present in both questionnaires, as coping for anxiety is enclosed within the coping ability.

The goal of the current study was a third step, to see if the PCDEQ-2 needed a new factor structure when assessed in a younger age group. The EFA has shown that a 5-factor structure, with less items, indeed provides a better fit for this younger sample. However, the 5-factor structure is still based upon the 7-factor structure, with factors that are coming back in the PCDEQ-2 (questionnaire for the older age group), as can be seen in the table below. This is now also included as a supplementary material.

Lines 252-255: In naming each factor, we followed the usual convention of focusing on the content (28, 33). As a secondary consideration, however, we tried to stay as close as possible to the factor names selected for the PCDEQ2 (1) (S3 Table).

| **S3 Table.** PCDE factors per age category. | | |
| --- | --- | --- |
|  | **PCDEQ-C** | **PCDEQ-2** |
| **Factor 1** | Adverse Response to Failure (-) | Adverse Response to Failure (-) |
| **Factor 2** | Imagery and Active Preparation (+) | Imagery and Active Preparation (+) |
| **Factor 3** | Self-Directed Control and Management (+) | Self-Directed Control and Management (+) |
| **Factor 4** | Performance Worries (-) | Perfectionistic Tendencies (+/-) |
| **Factor 5** | Seeking and Using Social Support (+) | Seeking and Using Social Support (+) |
| **Factor 6** |  | Active Coping (+) |
| **Factor 7** |  | Clinical Indicators (-) |

References:

- MacNamara, Á., & Collins, D. (2011). Development and initial validation of the psychological characteristics of developing excellence questionnaire. Journal of Sports Sciences, 29(12), 1273-1286.
- Hill, A., MacNamara, Á., & Collins, D. (2019). Development and initial validation of the Psychological Characteristics of Developing Excellence Questionnaire version 2 (PCDEQ2). European journal of sport science, 19(4), 517-528.

The Imaginary factor is not well understood in 7-year-olds. Self-assessment of visualization ability in a 7-year-old and a 13-year-old cannot be equipped. What level of control and clarity does the 7-year-old have to visualize a game or training action?

We thank the reviewer for his concern, although we do believe that the use of imagery is extremely common in younger children (Munroe-Chandler et al., 2007). Young children use imagery abilities very common in daily life activities (e.g., by playing little games as ‘hide and seek’, ‘pretending to be a policeman’, etc.). During childhood, the children will ill develop and improve their imagery abilities from perceptual to conceptual activity (Forisha, 1975).

In an athlete context, coaches often use imagery to coach young children and clarify or highlight some required technical skills (e.g. “run as fast as a cheetah”; “imagine you are as strong as a bear”). Moreover, the review of Munroe-Chandler and colleagues (2007) has pointed out that athletes from the age of seven years old use imagery control during practice for both cognitive and motivational purposes. Other studies even found that young athletes are able to reflect upon their imagery use (Parker and Lovell, 2012; Di Corrado et al., 2020). This is further explained on the following lines:

Lines 90-98: Furthermore, research has already shown that, although young children might not comprehend the terms used in questionnaires or by experts, this does not mean they are not using the underlying psychological characteristics already in their athletic career. An example for this is imagery. Imagery is a difficult term to understand without explanation, but the skill is frequently used, although perhaps with differences in vividness and underlying cognitive abilities, by young athletes (18-20). This reflects a possible developmental difference in the knowledge, understanding and use of the psychological skills which would benefit from empirical examination and, if appropriate, remediation.

References:

- Parker, J. K., & Lovell, G. P. (2012). Age differences in the vividness of youth sport performers’ imagery ability. Journal of Imagery Research in Sport and Physical Activity, 7(1).
- Di Corrado, D., Guarnera, M., Guerrera, C. S., Maldonato, N. M., Di Nuovo, S., Castellano, S., & Coco, M. (2020). Mental imagery skills in competitive young athletes and non-athletes. Frontiers in psychology, 11, 633.
- Munroe-Chandler, K. J., Hall, C. R., Fishburne, G. J., & Strachan, L. (2007). Where, when, and why young athletes use imagery: An examination of developmental differences. Research quarterly for exercise and sport, 78(2), 103-116.

It is not clear how many compete, at what level they compete, and what performance is achieved.

We thank the reviewer for pointing this out, and we have now included the information available in a table in supplementary material.

Lines 179-182: This resulted in a total sample of 773 participants (400 girls), with 495 individual athletes (e.g. gymnastics, cycling and badminton) and 278 team sports (e.g. soccer and hockey) athletes. A detailed overview of the distribution per age, sex, type of sport and level can be found in S1 Table.

| **S1 Table**. Distribution per age and sex, type of sport and level. | | | | | | | | | | | |
| --- | --- | --- | --- | --- | --- | --- | --- | --- | --- | --- | --- |
|  |  | **Team sports** | | | **Individual sports** | | | **Total** | |  |  |
| **Age** | **Sex** | **Recreational** | **Competitive** | **Total** | **Recreational** | **Competitive** | **Total** | |  | |  |
| **7** | **Male** | 1 | 14 | 15 | 10 | 4 | 14 | | 29 | |  |
|  | **Female** | 1 | 3 | 4 | 18 | 0 | 18 | | 22 | |  |
| **8** | **Male** | 4 | 13 | 17 | 5 | 11 | 16 | | 33 | |  |
|  | **Female** | 0 | 1 | 1 | 23 | 34 | 57 | | 58 | |  |
| **9** | **Male** | 7 | 24 | 31 | 12 | 10 | 22 | | 53 | |  |
|  | **Female** | 5 | 7 | 12 | 25 | 46 | 71 | | 83 | |  |
| **10** | **Male** | 7 | 38 | 45 | 8 | 19 | 27 | | 72 | |  |
|  | **Female** | 5 | 6 | 11 | 35 | 30 | 65 | | 76 | |  |
| **11** | **Male** | 10 | 39 | 49 | 7 | 32 | 39 | | 88 | |  |
|  | **Female** | 3 | 9 | 12 | 19 | 28 | 47 | | 59 | |  |
| **12** | **Male** | 8 | 48 | 56 | 6 | 36 | 42 | | 98 | |  |
|  | **Female** | 8 | 17 | 34 | 34 | 43 | 77 | | 102 | |  |

Since this is only limited sociodemographic information, we do agree with the reviewer that incorporating information on sporting achievements would be valuable in the future. We added this comment to the limitation section:

Lines 403-410: Since this was only a first step in the validation process, further research is necessary. Further work should investigate the confirmation of the proposed factor structure, in the different contexts as suggested, and with large sample sizes. More types of sport should be included, so future work could also investigate the discriminating ability in type of sport. Other information regarding training history, training intensity, competitive achievements and/or other sociodemographic variables should also be explored.

The administration of questionnaires to gymnastics and soccer participants is not representative of the rest of the sports (there are not combat sports; there are not racket sports; there are not sports such as athletics).

The questionnaire was not solely restricted to be filled out by gymnasts and soccer athletes, we also used athletes from other sports to validate the questionnaire (e.g., hockey, triathlon, cycling, ..).

However, we do agree with the reviewer that more types of sport should be included in future research. One example could be to identify differences in how athletes deploy the PCDEs in different types of sport. We have included this in the limitation section.

Lines 403-408: Since this was only a first step in the validation process, further research is necessary. Further work should investigate the confirmation of the proposed factor structure, in the different contexts as suggested, and with large sample sizes. More types of sport should be included, so future work could also investigate the discriminating ability in type of sport.

Two weeks in the test-retest is a very short period of time. The adapted questionnaire is a questionnaire based on a perception of the situational athlete. This data makes it difficult to establish long-term performance predictions.

A two-week period is long enough if the purpose is a test-retest procedure. It is indeed true that some studies use longer test-retest procedures as for example Kline (1998) advises, some studies also use only one week between both test occasions (Crocker et al., 1997; Lane et al., 2005). For the current study, since this is indeed a self-report and self-perception type of questionnaire, a longer test-retest period was not advocated and the time between two test moments was reduced (in agreement with Cruise et al., 2006 or Uphill et al., 2012). This to avoid causing changes in the athletes’ perceptions over time (Koole et al., 2010). We argue on these lines not to have a too lengthy interval between two test occasions:

Lines 288-291: The interval between the test-retest moments should not be too small, because the participants could still remember their answers, but it should also not be too large, so that the conditions were still comparable (39) and athlete’s perceptions would not change (40). Accordingly, we decided that participants completed the questionnaire twice, with a two-week rest period in between.

As is previously said, the PCDEQ-C is not a selection tool, hence it is not advised to use this questionnaire for long-term predictions. The PCDEQ-C can be used as a form to base psycho-behavioural interventions, which can help the athlete successfully and effectively progress through the talent development pathway.

References:

- Kline, R. B. (2015). Principles and practice of structural equation modeling. Guilford publications.
- Crocker, P. R., Bailey, D. A., Faulkner, R. A., Kowalski, K. C., & McGrath, R. (1997). Measuring general levels of physical activity: preliminary evidence for the Physical Activity Questionnaire for Older Children. Medicine and science in sports and exercise, 29(10), 1344-1349.
- Lane, A. M., Nevill, A. M., Bowes, N., & Fox, K. R. (2005). Test-retest stability of the Task and Ego Orientation Questionnaire. Research Quarterly for Exercise and Sport, 76(3), 339-346.
- Cruise, S. M., Lewis, C. A., & Guckin, C. M. (2006). Internal consistency, reliability, and temporal stability of the Oxford happiness questionnaire short-form: Test-retest data over two weeks. Social Behavior and Personality: an international journal, 34(2), 123-126.
- Uphill, M. A., Lane, A. M., & Jones, M. V. (2012). Emotion Regulation Questionnaire for use with athletes. Psychology of Sport and Exercise, 13(6), 761-770.
- Koole, S. L., Van Dillen, L. F., & Sheppes, G. (2011). The self-regulation of emotion. Handbook of self-regulation: Research, theory, and applications, 2, 22-40.

Why is the Omega reliability index used and not the Cronbach's Alpha coefficient?

Both Cronbach’s alpha and omega are good reliability measures. However, as is pointed out by Revelle and Zinbarg (2009), Cronbach’s alpha is based on very strict assumptions (data has to be tau-equivalent and factors have to be unidimensional), assumptions that are rarely met in research studies. Therefore, there is a shift from Cronbach’s alpha to omega. According to Revelle and Zinbarg (2009), omega is a more precise measure of reliability for both unidimensional and multidimensional measures and it gives the ‘greatest lower bound of reliability’. Furthermore, omega value is also more robust for factors with a low number of items loading onto it; whereas Cronbach’s alpha sometimes underestimates the reliability in this case (Pallant, 2011).

However, as the reviewer can see, the Cronbach’s alpha are very similar to the omega value reported in this study:

|  | Factor 1 | Factor 2 | Factor 3 | Factor 4 | Factor 5 |
| --- | --- | --- | --- | --- | --- |
| Cronbach’s alpha | 0.87 | 0.73 | 0.85 | 0.78 | 0.66 |
| McDonald omega | 0.86 | 0.73 | 0.84 | 0.75 | 0.65 |

References:

- Zinbarg, R., Revelle, W., Yovel, I., & Li, W. (2005). Cronbach’s α, revelle’s β, and mcdonald’s ωh: Their relations with each other and two alternative conceptualizations of reliability. Psychometrika, 70(1), 123–133.
- Pallant, J. (2011). SPSS survival manual: A step by step guide to data analysis using SPSS (4th ed.). Maidenhead, Australia: Open University Press/McGraw-Hill.

Test-retest correlations obtained below .70 are not adequate. They are small, and should be considered a limitation in the study. This data indicates the low temporal stability of the scores obtained, limiting the ability to establish medium and long-term predictions. With test-retest administrations lasting approximately one month, it is very likely that the correlations were much lower.

With a good overall omega, some specific factors receiving a good classification and others a lower classification, we believe the overall reliability is considered adequate. We based our classification upon previous research, as is explained here:

Lines 300-302: McDonald omega value was used as a measure for internal consistency within and between the factors. Omega values can range between 0 and 1, where a value closer to 1 and above 0.70 is considered to represent a good reliability (41, 42).

However, we have made changes in the manuscript, to soften the tone.

Lines 311-312: For factors 2 and 5, correlations between 0.60 and 0.68 were seen, considered as rather weak correlations.

We have indeed added some lines on the reliability in the limitation section.

Lines 399-402: Lastly, although overall reliability is considered adequate, low reliability scores were observed on two factors. When follow-up is provided on the results of the PCDEQ-C, special attention should be given to these two factors.

Nevertheless, the PCDEQ-C is a tool that should be used as a formative assessment tool, that gives an indication about the psycho-behavioural skills an athlete is using at that moment in his talent development process. Since not only the talent development pathway, but also the psycho-behavioural development is non-linear and dynamical, these sorts of questionnaires should not be used as a (long-term) prediction or selection tool. To predict future success, other types of assessment (e.g. motor coordination or competition results) should be used. But even here prediction scores are relatively low, as, again, development is non-linear, dynamic, and thus changeable.

DISCUSSION

Further development of the limitations of the study is lacking.

Limitations are addressed at lines 393-403. We have now added that this is indeed a self-report questionnaire, and that reliability scores on two factors were low.

Lines 392-402: Some limitations regarding the validation process of the PCDEQ-C should be addressed. First, the PCDEQ-C is now based on age-cut offs, although it could be interesting to investigate if cognitive maturity level could be a better indicator for understanding and using the PCDEs. It should also be recognised that the questionnaires regarding the PCDEs are filled-in by the athletes themselves. Next to the general issues with self-report (ref), special care should be given to the competitive nature of talent development environments, where individuals potentially employ impression management strategies when responding to any questionnaire. Lastly, although overall reliability is considered adequate, low reliability scores were observed on two factors. When follow-up is provided on the results of the PCDEQ-C, special attention should be given to these two factors.

Further development of the practical implications of the study for sports coaches and psychologists is lacking.

We have now tried to clarify the practical implications in the conclusion:

Lines 424-431: Rather than a talent selection tool, this questionnaire should be used as a formative assessment tool to monitor, develop, and design the psychological characteristics needed during the entire TDE pathway of a young, potential athlete. The questionnaire is being increasingly reported as a practical and comprehensive tool to measure PCDEs by both athletes and coaches (45) and should be used as part of the triangulation process. The use of other measures, such as behavioural observations, coaches’ opinions, dialogue with family or guardians, and follow-up of the PCDEQ-C results by coaches and/or sport psychologist are advised.

Further development of future research lines to follow is lacking.

We thank the reviewer of pointing this out and have now addressed this in the discussion.

Lines: 403-421: Since this was only a first step in the validation process, further research is necessary. Further work should investigate the confirmation of the proposed factor structure, in the different contexts as suggested, and with large sample sizes. More types of sport should be included, so future work could also investigate the discriminating ability in type of sport. Other information regarding training history, training intensity, competitive achievements and/or other sociodemographic variables should also be explored. Next the application of the PCDEs in these younger ages also needs further attention, especially against self-presentational bias. An example thereof is to investigate to what extent the answers the young children gave are in line with what parents or coaches observe; in short, to evaluate and confirm the self-reflection capacity of young children. Finally, this study is also cross-sectional in nature. Longitudinal study designs are necessary, to follow-up of the development of the PCDE’s and gain knowledge of the psychological skills and characteristics that contribute to specific stages of the talent development process.

Important bibliographic references are missing in the paper.

Without more specific input we are unable to address this comment. However, we are confident that sufficient references are offered and can see no arguments that would benefit from such additionally.

Differences in scores have not been established based on age, sport age category, performance level and category by sex. The discriminatory ability of the test cannot be determined.

We agree with the reviewer and this was also not the scope of this article. The current study only wanted to initially validate the PCDEQ-C, by exploring the factor structure. However, future research should investigate the differences or discriminatory ability of the PCDEQ; especially to see if there are differences in how different types of athletes deploy the PCDEs at young ages.

Lines 370-391: To further validate the PCDEQ-C, more information about the understanding and application of PCDEs in different contexts is necessary. Planning and organisation, one of the PCDEs, is already of importance during childhood. However, younger athletes will likely rely on their parents and coaches for their planning, without giving it further thought. At a later stage, when athletes are older and further down their talent development pathway, they will make the planning in co-creation with the coach (42). Next to age, differences in sex in these younger age groups needs further examination. There are for example sex-based differences in the prevalence of certain psychopathological problems in adolescent athletes, such as eating or anxiety disorders, two maladaptive characteristics also used in the current study (43). Sex differences may also covary with maturation, as girls at this stage may often be ahead of boys both physically and emotionally (44). Examining the differences between young boys and girls on not only these maladaptive, but also positive psychological characteristics, is thus necessary. Coinciding with age- and sex differences, therefor another factor that needs recognition, is the type of sports young athletes participate in. Next to the typical differences in team sports vs individual sports, already seen in the adolescent age group (6), athletes from the same age could be in different talent developmental stages, depending on their sport. Early specialisation sports (e.g. swimming, gymnastics,..) require a higher and more complex set of (psychological) skills at younger ages than normal or late specialisation sports (e.g. badminton, soccer, …). At the moment, only age is considered in the development of the PCDEs, although perhaps more information will come when approaching the PCDEs according to the talent development stage.

This is also further addressed in the future research section, were we also added more information:

Lines 405-408: Further work should investigate the confirmation of the proposed factor structure, in the different contexts as suggested, and with large sample sizes. More types of sport should be included, so future work could also investigate the discriminating ability in type of sport.

It is not understood why the test has been administered to athletes aged less than 11-12 years. There are basic theoretical and methodological factors that indicate the inappropriateness of administering self-report questionnaires with ages less than 12 years.

We thank the reviewer for his concern, but we believe a simple age cut-off is not the (only) way to identify the ‘inappropriate’ participants. We are aware of the young ages we are using in the current paper, and we have tried to address this.

Methodologically, steps were taken to make sure the items were age-appropriate. We also advise to administer the questionnaire in a quiet room, with a one-on-one guidance by an independent adult for the younger athletes.

Lines 135-138: First, the appropriateness of the English items in the original questionnaire (the PCDEQ2) was checked by the Belgian-British panel. Items that were too difficult for 8-year-olds were rephrased in English. This only led to rewriting 3 items, specifically because of the term ‘visualisation’.

Lines 365-369: During testing, it was observed that filling-out the questionnaire was difficult for the youngest participants. Although they understood the items in the questionnaire, they were more comfortable providing answers when a researcher read the items out loud. We advise to complete the assessment of the PCDEQ-C in a quiet room, with a one-on-one guidance, to make the responses of the young athletes as accurate as possible.

Furthermore, we emphasized the need to further examine the influence of cognitive and biological maturity and the self-reflection abilities of the young athletes in the discussion section.

Lines 393-395: First, the PCDEQ-C is now based on age-cut offs, although it could be interesting to investigate if cognitive maturity level could be a better indicator for understanding and using the PCDEs.

Lines 410-414: Next the application of the PCDEs in these younger ages also needs further attention, especially against self-presentational bias. An example thereof is to investigate to what extent the answers the young children gave are in line with what parents or coaches observe; in short, to evaluate and confirm the self-reflection capacity of young children.

It is not understood why psychopathological factors are introduced when the test does not measure psychopathological constructs or other psychopathological questionnaires have been administered.

The PCDEQ-C is a self-perception assessment that maps psychological behaviour and indeed not psychological factors. If for example an athlete points out that he/she is concerned about food or body image, this does not directly mean the athlete has an eating disorder. It does however flag maladaptive psychological behaviour, which should be followed-up with other instruments by professionals. That is why the PCDEQ-C should be part of a triangulation process, which we have now added in the discussion:

Lines 424-431: Rather than a talent selection tool, this questionnaire should be used as a formative assessment tool to monitor, develop and design the psychological characteristics needed during the entire TDE pathway of a young, potential athlete. The questionnaire is being increasingly reported as a practical and comprehensive tool to measure PCDEs by both athletes and coaches (45) and should be used as part of the triangulation process. The use of other measures, such as behavioural observations, coaches’ opinions, dialogue with family or guardians, and follow-up of the PCDEQ-C results by coaches and/or sport psychologist are advised.

It is noteworthy how the authors, knowing the importance of the evolution of talent and not talent at a specific moment, have not carried out a longitudinal study (with fewer participants and with a relatively short time).

We agree with the reviewer that longitudinal studies should be carried out, as we included in the discussion:

Lines 418-421: Longitudinal study designs are necessary to follow-up of the development of the PCDE’s and gain knowledge of the psychological skills and characteristics that contribute to specific stages of the talent development process.

However, first a validated instrument is needed, than we can follow up longitudinally with that same instrument. The current study thus took a first step in the validating the instrument. Now, further avenues can be followed for research with the PCDEQ-C. Future research lines are now added to a new paragraph:

Lines 403-410: Since this was only a first step in the validation process, further research is necessary. Further work should investigate the confirmation of the proposed factor structure, in the different contexts as suggested, and with large sample sizes. More types of sport should be included, so future work could also investigate the discriminating ability in type of sport. Other information regarding training history, training intensity, competitive achievements and/or other sociodemographic variables should also be explored.

Why is the factorial structure not maintained with respect to the previous tests?

We think the reviewer is asking whether we did not use a CFA with the 7-factor structure proposed by Hill and colleagues (2019) and see if this would give a good fit for the current sample.

We explicitly choose not to use the 7-factor structure with a CFA, because we hypothesised that a different factor structure would be needed for the younger athletes. That is why we chose to use EFA in this study and explore which items and factor structure would be needed in the young age group. We explained our rationale in the introduction, for example here:

Lines 103-110: For psychological skills, athletes will be exposed to different needs in the initiation, development and mastery stage (5). As athletes continue their developmental process, the demands of the environment become more difficult or change in type and balance. Consequently, although the fundamentals of the PCDEs could be present in the preadolescent group, not all PCDEs may be as valuable or meaningful as in the older age group, therefore adding to the potential for a different factor structure in the preadolescent athletes.

The EFA indeed pointed out that the 7-factor solution was not a good fit for our younger sample. This result, coupled with much of the literature cited and in the area, suggests that the factor structure seems to change over age, going from a 5-factor to a 7-factor structure.

L341-343: The 5 factors proposed here, suggests that coherence can change over age, going from a 5-factor structure in childhood athletes to a 7-factor structure in adolescent athletes (Hill et al., 2019).

References:

- Hill, A., MacNamara, Á., & Collins, D. (2019). Development and initial validation of the Psychological Characteristics of Developing Excellence Questionnaire version 2 (PCDEQ2). European journal of sport science, 19(4), 517-528.

The implications of the test for a sports psychologist are not included at any time, and yet psychopathological terms are included.

The psychopathological terms included in this questionnaire are solely to alert the athlete, coach and/or involved sport psychologist that the athlete is using maladaptive behaviour at that moment in his talent development process. Of course, as the PCDEQ-C is a formative assessment tool, this should be further addressed with athlete, coach and sport psychologist. We have now added this in the conclusion section, that a triangulation process is indeed necessary.

Lines 424-431: Rather than a talent selection tool, this questionnaire should be used as a formative assessment tool to monitor, develop, and design the psychological characteristics needed during the entire TDE pathway of a young, potential athlete. The questionnaire is being increasingly reported as a practical and comprehensive tool to measure PCDEs by both athletes and coaches (45) and should be used as part of the triangulation process. The use of other measures, such as behavioural observations, coaches’ opinions, dialogue with family or guardians, and follow-up of the PCDEQ-C results by coaches and/or sport psychologist are advised.

The reviewer considers that for the publication of the article the following should be included:
1.- The initial and final test items.

We have now added the PCDEQ-C in a supplementary file, and not the PCDEQ-2. The initial items (from the PCDEQ-2) can be found in the article from Hill and colleagues (2019), which we clarified in the method section.

Lines 130-132: The original PCDEQ2 focused on 14-to-19-year-old male athletes in team sports and needed to be adapted to 7-to-13-year-old male and female athletes. A list of the factors and the corresponding items can be found in the article of Hill et al. (2019).

Lines 234-236: From the 65 items originally in the EFA model, 12 items with an item loading under 0.3 were removed (25). This model ended with 53 items corresponding to 5 factors (Table 3 and S2 Table).

References:

- Hill, A., MacNamara, Á., & Collins, D. (2019). Development and initial validation of the Psychological Characteristics of Developing Excellence Questionnaire version 2 (PCDEQ2). European journal of sport science, 19(4), 517-528.

2.- With such a high number of athletes evaluated, it would be advisable to include the corresponding scales.

We are not sure what the reviewer means with ‘corresponding scales’, however if it regards the Likert scale used in this study, this information is included on these lines:

Lines 187-189: The questionnaire consisted of 87 items on a 6-point Likert scale, ranging from 1 (“very unlike me”) to 6 (“very like me”).

If this is not the information the reviewer wanted, is it be possible to be more explicit, so that these data could be added to the supplementary material.

6. **Do you want your identity to be public for this peer review?**

Reviewer #1: **Yes:** Roberto Ruiz-Barquín (Universidad Autónoma de Madrid; Spain)
